# Correlates of Skilled Nursing Facility (SNF) Performance in the SNF Value-Based Purchasing Program

**DOI:** 10.1093/geroni/igaa057.273

**Published:** 2020-12-16

**Authors:** Dan Andersen, Sherly Binu, Mike Sacca

**Affiliations:** 1 RELI Group, Sykesville, Maryland, United States; 2 RELI Group, Baltimore, Maryland, United States; 3 RELI Group, Baltimore, United States

## Abstract

We examined the results of the 2020 skilled nursing facility (SNF) value-based purchasing (SNF VBP) program to identify correlates and potential drivers of SNF performance in this program. The SNF VBP program provides incentive payments to SNFs based on their performance on a risk-adjusted hospital readmission measure (i.e., the rate at which SNF residents are admitted back to the hospital within 30-days of being admitted to the SNF). SNFs are assessed on this measure for both improvement compared to their historical baseline and overall achievement compared to their peers. All SNFs that are covered under Medicare’s prospective payment system are included in the SNF VBP program. We performed analyses to assess the correlation between individual SNFs’ performance in the 2020 SNF VBP (n=15,201), which is based on actual performance in fiscal year 2018, with contemporaneous matched data related to SNF health inspection results, staffing, and performance on quality measures (these data form the basis of the five-star quality rating system on the Nursing Home Compare website). We also examined longitudinal trends in these non-SNF VBP program variables and their association with changes in SNF performance in the SNF VBP program. We controlled for important SNF-specific factors (e.g., for-profit status, connected to a hospital). We found strong contemporaneous and longitudinal associations between SNF VBP program performance and some, but not all, of these factors. Our findings are supported by decades of empirical research in SNF quality and highlight potential policy alternatives that could further incentivize high quality care in SNFs.

